# Data on estimations of relative dose rates along central axis of symmetric and asymmetric ${}^{106}$Ru/${}^{106}$Rh applicators used in eye brachytherapy

**DOI:** 10.1016/j.dib.2020.106620

**Published:** 2020-12-06

**Authors:** Eduardo De Paiva

**Affiliations:** Division of Medical Physics, Institute of Radiation Protection and Dosimetry – IRD/CNENAv. Salvador Allende 3773, 22783-127 Rio de Janeiro, Brazil

**Keywords:** Beta particles, ophthalmic applicators, symmetric ${}^{106}$Ru/${}^{106}$Rh plaques, asymmetric ${}^{106}$Ru/${}^{106}$Rh plaques

## Abstract

Beta particles radiation doses have important applications in medicine. In particular, curved and symmetric as well as curved and asymmetric applicators containing the beta emitting ${}^{106}$Ru/${}^{106}$Rh isotopes are widely used in radiotherapy for the treatment of various ocular diseases. Nevertheless, a great problem in the use of these applicators is the inaccurate determination of the dose rates around them. Difficulties arise mainly because of the very short distances involved, and in this scenario theoretical calculation methods play an important role. In this work a simple approach based on the beta-point dose function integration over the total surface of each plaque was used to estimate the dose rates along their central axis. Results of relative dose rates for concave and symmetric (CCA, CCB, CXS, CCX/Y/Z, CCD, CGD and CCC) and concave and asymmetric (CIA, CIB/CIB-2, COB and COC) ruthenium/rhodium plaque types are shown.

## Specifications Table

**Table:** 

Subject	Medical physics
Specific subject area	Radiation therapy, brachytherapy
Type of data	Tables
How data were acquired	Numerical integration of the beta-point dose function over the radioactive surface of the plaques
Data format	Raw
Parameters for data collection	Data presented are formed by depths versus relative dose rates for concave and symmetric (CCA, CCB, CXS, CCX/Y/Z, CCD, CGD and CCC) and concave and asymmetric (CIA, CIB/CIB-2, COB and COC) ruthenium/rhodium model applicators
Description of data collection	A Fortran code was developed to carry out numerical calculations for each of the 14 plaques analysed. For the asymmetric plaques an IF command makes the compiler to skip the points within the cut-out section
Data source location	Division of Medical Physics, Institute of Radiation Protection and Dosimetry (Rio de Janeiro, Brazil)
Data accessibility	Data provided within this article
Related research articles	E. De Paiva. Estimates of relative beta radiation doses on central and lateral axes of ruthenium/rhodium COB-type plaque used in eye brachytherapy. Applied Radiation and Isotopes 156, 108991 (2020). doi:10.1016/j.apradiso.2019.108991

## Value of the Data

• Symmetric and asymmetric applicators containing the ${}^{106}$Ru/${}^{106}$Rh beta emitters play an important role in radiotherapy to treat various diseases. However, mainly because of the short distances involved, there is a scarcity of dosimetric data on them, specially for the asymmetric plaques. Therefore, all dataset on dose rates around these plaques are welcome and makes the results shown here valuable.
• Users from small radiotherapy facilities, mainly located in low-income and developing countries where computational resources may be limited, can take advantage of these rough estimates to plan doses around these types of applicators.
• Due to the great lack of published experimental and theoretical data on these kinds of applicators the results presented here, mainly for the asymmetric plaques, may be used as a reference for future studies on them.
• On the clinical usability of the data we highlight that the dataset can be used as input in the developing of a treatment planning system; users/researches around the world may also develop a software to estimate the doses around these beta applicators and results can be compared to the data presented.

## 1 Data Description

Some details on the plaques studied are shown in Table 1. In Table 2 are shown the dose rates as a function of depths along central axis of the plaques. Results are normalised to 100% at 1 mm depth on the central axis of the concave and symmetric ruthenium/rhodium CCA, CCB [1] and CXS, CCX/Y/Z, CCD, CGD, CCC model plaques.

**Table 1:** Applications and dimensions of the ruthenium/rhodium plaques analysed.

Plaque	Medical	Radius of	Active
type	application	curvature [mm]	diameter [mm]
CXS	Retinoblastoma	12	7.7
CCX/Y/Z	Retinoblastoma	12	9.5
CCA	Uveal and choroidal melanomas	12	13
CIA	Melanomas close to the iris	12	13
CCD	Uveal and choroidal melanomas	12	15.5
COB	Tumours close to the optical nerve	12	17.1
CCB	Uveal and choroidal melanomas	12	17.8
CIB/CIB-2	Melanomas close to the iris	12	17.8
CGD	Uveal and choroidal melanomas	13	19.9
CCC	Uveal and choroidal melanomas	13	22.5
COC	Tumours close to the optical nerve	14	22.7

**Table 2:** Relative depth-doses for the curved and symmetric ${}^{106}$Ru/${}^{106}$Rh model applicators. Depths increase from the plaque center.

	Relative dose rates						
depth [mm]	CXS	CCX/Y/Z	CCA	CCD	CCB	CGD	CCC
0.5	1.443	1.390	1.338	1.319	1.309	1.309	1.306
1.0	1.000	1.000	1.000	1.000	1.000	1.000	1.000
1.5	0.750	0.775	0.804	0.815	0.821	0.821	0.823
2.0	0.578	0.616	0.662	0.681	0.692	0.692	0.696
2.5	0.450	0.493	0.548	0.573	0.588	0.588	0.594
3.0	0.350	0.393	0.453	0.481	0.498	0.499	0.507
3.5	0.271	0.312	0.372	0.402	0.422	0.423	0.432
4.0	0.210	0.246	0.304	0.335	0.356	0.358	0.368
4.5	0.162	0.194	0.248	0.278	0.299	0.302	0.313
5.0	0.125	0.152	0.200	0.229	0.250	0.254	0.265
5.5	0.096	0.119	0.161	0.188	0.209	0.212	0.224
6.0	0.074	0.093	0.130	0.154	0.173	0.177	0.189
6.5	0.057	0.073	0.104	0.125	0.143	0.147	0.159
7.0	0.044	0.056	0.082	0.101	0.117	0.121	0.133
7.5	0.034	0.044	0.065	0.081	0.096	0.100	0.111
8.0	0.026	0.034	0.051	0.065	0.078	0.081	0.092
8.5	0.020	0.026	0.040	0.051	0.062	0.066	0.075
9.0	0.015	0.020	0.031	0.040	0.050	0.053	0.062
9.5	0.011	0.015	0.024	0.031	0.039	0.042	0.050
10.0	0.008	0.011	0.018	0.024	0.031	0.033	0.040
10.5	0.006	0.008	0.014	0.018	0.024	0.026	0.032
11.0	0.004	0.006	0.010	0.014	0.018	0.020	0.025
11.5	0.003	0.004	0.007	0.010	0.013	0.015	0.019
12.0	0.002	0.003	0.005	0.007	0.010	0.011	0.014
12.5	-	-	-	-	-	0.007	0.010
13.0	-	-	-	-	-	0.005	0.007

In Table 3 are shown the dose rates as a function of depths along central axis of the plaques. Results are normalised to 100% at 1 mm depth on the central axis of the concave and asymmetric ruthenium/rhodium CIA, CIB/CIB-2, COB [2] and COC model plaques.

**Table 3:** Relative depth-doses for the curved and asymmetric ${}^{106}$Ru/${}^{106}$Rh model applicators. Depths increase from the plaque center.

	Relative dose rates			
depth [mm]	CIA	COB	CIB/CIB-2	COC
0.5	1.402	1.339	1.360	1.367
1.0	1.000	1.000	1.000	1.000
1.5	0.777	0.807	0.797	0.802
2.0	0.625	0.700	0.656	0.668
2.5	0.510	0.562	0.546	0.564
3.0	0.415	0.471	0.456	0.478
3.5	0.337	0.395	0.380	0.405
4.0	0.273	0.330	0.316	0.343
4.5	0.221	0.275	0.263	0.291
5.0	0.178	0.228	0.218	0.245
5.5	0.143	0.189	0.180	0.207
6.0	0.114	0.155	0.148	0.174
6.5	0.091	0.127	0.122	0.145
7.0	0.072	0.104	0.099	0.121
7.5	0.056	0.084	0.081	0.100
8.0	0.044	0.068	0.065	0.083
8.5	0.034	0.054	0.052	0.068
9.0	0.027	0.043	0.041	0.055
9.5	0.020	0.034	0.033	0.044
10.0	0.016	0.026	0.026	0.035
10.5	0.012	0.020	0.020	0.027
11.0	0.009	0.015	0.015	0.021
11.5	0.006	0.011	0.011	0.016
12.0	0.004	0.008	0.008	0.011
12.5	-	-	-	0.008
13.0	-	-	-	0.005
13.5	-	-	-	0.003
14.0	-	-	-	0.001

## 2 Methods

The relative depth-doses for the curved and symmetric and curved and asymmetric ${}^{106}$Ru/${}^{106}$Rh plaques were obtained by the following integral, evaluated over the plaque surface S,

(1) $$\dot{D}=a_{S}\int_{S}J\left(\xi \right)\cdot dS,$$

where $\dot{D}$ is the absorbed dose rate at the point of calculation, $a_{S}$ is the total activity per area and J(ξ), known as the beta-point dose function, is the dose rate at a distance ξ from a point-source on the plaque to the point of interest and is expressed by [3], [4]

(2) $$\begin{array}{ccc} J\left(\xi \right) & = & \frac{B}{\left(\rho \nu \xi \right){}^{2}}\{c\left[1-\frac{\rho \nu \xi }{c}\exp\left(1-\frac{\rho \nu \xi }{c}\right)\right]+\\ & & \rho \nu \xi \exp\left(1-\rho \nu \xi \right)-\rho \nu \xi \exp\left(1-\frac{\rho \nu \xi }{2}-\frac{f}{2}\right)\}, \end{array}$$

where ρ is the density of the absorbing medium, ν is the apparent absorption coefficient, and c and f are dimensionless parameters. The factor B is a normalization constant given by

(3) $$B=0.046\rho^{2}\nu^{3}{\bar{E}}_{\beta }\alpha ,$$

where ${\bar{E}}_{\beta }$ is the mean kinetic energy of the beta particles, and the factor α is related to parameters c and f as

(4) $$\frac{1}{\alpha }=3c^{2}-\left(c^{2}-1\right)\exp\left(1\right)+\left(3+f\right)\exp\left(1-f\right)-4\exp\left(1-\frac{f}{2}\right).$$

In the integration, through Eqs. (1) to (4), let us initially suppose that all the plaques are symmetric, i.e., the cut-out section does not exist, so that using spherical coordinates we can write

(5) $$\dot{D}=a_{S}R^{2}\int \;\;\;\int J\left(\xi \right)\;\sin\phi \;d\phi \;d\theta ,$$

where R is the constant plaque radius of curvature; the angle θ is the azimuthal angle in the xy-plane from the x-axis; ϕ is the polar angle from the positive z-axis. We can easily determine the distance ξ from a point on the plaque to a point P(0,0,$z_{0}$) on the central axis as

(6) $$\xi =\sqrt{R^{2}+z_{0}^{2}-2Rz_{0}\cos\phi }.$$

The problem of the asymmetry of the actual plaques (CIA, CIB/CIB-2, COB, COC) can be solved by not considering the contribution of points on the source located inside the cut-out section. For every *point-source* inside the cut-out section an instruction makes the compiler to skip the calculations, and this is accomplished with an IF command within the code in order to skip the points in the xy-plane within the cut-out section.

The integration described above was carried out by means of a Fortran code based on the trapezoidal rule and the results of a such calculations (normalised to 100 % at 1 mm depth) are shown in Tables 2 and 3, respectively for curved and symmetric and curved and asymmetric ruthenium/rhodium plaques. These dataset can be used as input in the developing of a treatment planning system; users/researches around the world may also develop a software to estimate the doses around these beta applicators and results can be compared to the data presented. Users/researches interested in use the Fortran code can obtain it at the GitHub repository (https://github.com/edu2112923/Eduardo.git) or under request.

## Ethics Statement

No humans and animals were involved in the data collection.

## Declaration of Competing Interest

The author declares that he has no known competing financial interests or personal relationships which have or could be perceived to have influenced the work reported in this article.
